# A Three-Way Comparison of Tuberculin Skin Testing, QuantiFERON-TB Gold and T-SPOT.*TB* in Children

**DOI:** 10.1371/journal.pone.0002624

**Published:** 2008-07-09

**Authors:** Tom G. Connell, Nicole Ritz, Georgia A. Paxton, Jim P. Buttery, Nigel Curtis, Sarath C. Ranganathan

**Affiliations:** 1 Department of Paediatrics, University of Melbourne, Royal Children's Hospital Melbourne, Parkville, Victoria, Australia; 2 Murdoch Children's Research Institute, Royal Children's Hospital Melbourne, Parkville, Victoria, Australia; 3 Infectious Diseases Unit, Department of General Medicine, Royal Children's Hospital Melbourne, Parkville, Victoria, Australia; 4 Immigrant Health Service, Department of General Medicine, Royal Children's Hospital Melbourne, Parkville, Victoria, Australia; 5 Department of Thoracic Medicine Royal Children's Hospital Melbourne, Parkville, Victoria, Australia; University College London, United Kingdom

## Abstract

**Background:**

There are limited data comparing the performance of the two commercially available interferon gamma (IFN-γ) release assays (IGRAs) for the diagnosis of tuberculosis (TB) in children. We compared QuantiFERON-TB gold In Tube (QFT-IT), T-SPOT.*TB* and the tuberculin skin test (TST) in children at risk for latent TB infection or TB disease.

**Methods and Findings:**

The results of both IGRAs were compared with diagnosis assigned by TST-based criteria and assessed in relation to TB contact history. Results from the TST and at least one assay were available for 96 of 100 children. Agreement between QFT-IT and T-SPOT.*TB* was high (93% agreement, κ = 0.83). QFT-IT and T-SPOT.*TB* tests were positive in 8 (89%) and 9 (100%) children with suspected active TB disease. There was moderate agreement between TST and either QFT-IT (75%, κ = 0.50) or T-SPOT.*TB* (75%, κ = 0.51). Among 38 children with TST-defined latent TB infection, QFT-IT gold and T-SPOT.*TB* assays were positive in 47% and 39% respectively. Three TST-negative children were positive by at least one IGRA. Children with a TB contact were more likely than children without a TB contact to have a positive IGRA (QFT-IT LR 3.9; T-SPOT.*TB* LR 3.9) and a positive TST (LR 1.4). Multivariate linear regression analysis showed that the magnitude of both TST induration and IGRA IFN-γ responses was significantly influenced by TB contact history, but only the TST was influenced by age.

**Conclusions:**

Although a high level of agreement between the IGRAs was observed, they are commonly discordant with the TST. The correct interpretation of a negative assay in a child with a positive skin test in clinical practice remains challenging and highlights the need for longitudinal studies to determine the negative predictive value of IGRAs.

## Introduction

The detection and treatment of latent tuberculosis (TB) infection is a key strategy in the control of TB [1], [2]. Improved methods for detecting both latent TB infection and TB disease in children are needed. Interferon gamma release assays (IGRAs) incorporating *Mycobacterium tuberculosis* (MTB)-specific antigens have emerged as potential replacements for the century old tuberculin skin test (TST) [3]. IGRAs have been shown to have high sensitivity and specificity in adults [4] but there are few studies that have assessed their performance for the diagnosis of latent TB infection or TB disease specifically in children [5]. A recent meta-analysis highlighted an urgent need for more evidence for the use of IGRAs for the diagnosis of latent TB infection in children [6]. The aim of this study was to compare the performance of two commercial IGRAs with TST in the diagnosis of children at risk for latent TB infection or with suspected active TB disease at a tertiary paediatric hospital.

## Methods

### Patients and inclusion criteria

The study was approved by the Human Research and Ethics Committee of the Royal Children's Hospital Melbourne. Patients were recruited prospectively from the hospital's TB, refugee health and infectious diseases clinics. Children at risk of latent TB infection or with suspected active TB disease were eligible for inclusion. At risk was defined as a recent TB contact and/or recent immigration from a country with a high prevalence of TB. Written informed consent was obtained from parents or participants in their preferred language. Demographic and clinical details were obtained from each patient by a detailed questionnaire, including: country and date of birth; TB exposure history; Bacille Calmette-Guérin (BCG) vaccination history; presence of BCG scar and symptoms suggestive of TB. All patients had a full clinical evaluation, a TST and blood tests including IGRA, full blood count and CD4 lymphocyte count. HIV tests were not done as part of this study.

### Tuberculin skin test

A Mantoux test was performed by intradermal injection of 10 international units tuberculin (Purified Protein Derivative (PPD) 100 IU/mL, CSL, Melbourne, Australia), the standard dose of tuberculin PPD used in Australia at the time of the study, by trained personnel and read after 48 to 72 hours. An assessment of the level of risk for TB infection was taken into consideration when defining the result of the TST, as recommended by recently modified local guidelines (Victorian Department of Human Services). A positive TST was defined as ≥10 mm in patients with moderate risk factors (origin from high prevalence country; age 1 to 5 years); and ≥5 mm in patients with high risk factors (household TB contact; age less than 1 year). If BCG was given within five years prior to the TST, the TST was considered positive if induration was ≥15 mm in moderate risk patients and ≥10 mm in high risk patients. In children with suspected active TB disease, TST induration ≥5 mm was considered positive. Victorian guidelines for TST interpretation are similar to those of the American Thoracic Society (ATS), except that the potential influence of prior BCG is taken into consideration. Chest radiography was undertaken in those with a positive TST or when clinically indicated, and reported by radiologists who were blinded to the results of the IGRAs for each patient. Children deemed to have latent TB infection were offered isoniazid preventive treatment.

### Interferon gamma release assays

A whole blood assay, QuantiFERON-TB gold In-Tube (QFT-IT), Cellestis Ltd, Australia and an enzyme-linked immunospot assay (ELISPOT), T-SPOT.*TB*, Oxford Immunotec, Oxford, UK were carried out according to the manufacturers' guidelines and defined as positive, negative or indeterminate based on manufacturers' recommended criteria. The laboratory scientists undertaking the IGRAs were blinded to the clinical status of the patients. The QFT-IT (3^rd^ generation) assay incorporates an additional MTB-specific antigen TB 7.7 in addition to ESAT-6 and CFP-10. This assay was undertaken at the Victorian Infectious Disease Reference Laboratory Melbourne (VIDRL). The T-SPOT.*TB* test was undertaken in the Microbiology Research Laboratory at the Royal Children's Hospital Melbourne. Spots were counted with a magnifying glass and expressed as spots per million peripheral blood mononuclear cells (PBMC). Two further independent observers, also blinded to clinical status allocation, confirmed spot counts and assigned results.

### Definitions

*Latent TB infection* was defined as an asymptomatic child with a positive TST and chest radiograph not suggestive of TB. *Active TB disease* was defined as a child with a positive TST with symptoms suggestive of TB and/or an abnormal chest radiograph consistent with TB, or a child with MTB cultured from clinical specimens. Symptoms considered suggestive of TB included the following: cough for more than two weeks, persistent fever, night sweats and weight loss. *Uninfected* was defined as a well child with a negative TST or a child with symptoms potentially suggestive of TB but in whom results of all investigations for TB were negative or a child with an alternative diagnosis and complete recovery in the absence of specific TB treatment.

### Statistics

Data were analysed using Prism Graphpad (Version 5). Nonparametric unpaired data (TST indurations) were analysed by the Mann-Whitney U test. The mean age of children was compared using an unpaired t test or one-way anova. A Fisher's exact or chi-square test was used to compare proportions when the results of IGRAs were analysed as dichomatous outcomes, and an unpaired t test was used when IGRA antigen responses were analysed as continuous measures. Agreement between TST and IGRAs was assessed by the kappa statistic [7]. A multivariate linear regression model was used to determine the influence of age, BCG scar, origin from country with high TB prevalence and TB contact on the results of TST and IGRAs. Correlations were assessed using the Spearman's correlation coefficient.

## Results

Clinical and demographic details are shown in Table 1. Of 101 children enrolled in the study, four (4%) failed to return for a TST reading and could not be categorised according to the study criteria. *Mycobacterium gordanae* was cultured from the sputum of one child, who was subsequently excluded from further analysis. Of the remaining 96 children, 82 (85%) originated from a high TB prevalence country. On the basis of pre-defined criteria for categorising patients to diagnostic groups, 38 (40%) children had latent TB infection, 9 (9%) TB disease and 49 (51%) were uninfected. The mean age of the children in the three groups was not significantly different (p = 0.19). No child had a clinical presentation consistent with HIV infection and all children had a CD4% within the normal range for age.

**Table 1:** Demographic, clinical and laboratory details of study participants.

	Latent TB	TB disease	Uninfected
	(n = 38)	(n = 9)	(n = 49)
***Demographic***			
Mean age in years (range)	10.2 (0.7–18.8)	8.2 (1.8–13.6)	8.4 (0.5–19.0)
Male	17 (45%)	5 (55%)	28 (57%)
Born in high TB prevalence area	30 (79%)	8 (89%)	43 (88%)
TB contact			
Household	16 (42%)	8 (89%)	10 (20%)
Non-household	6 (16%)	1 (11%)	1 (2%)
No known contact	16 (42%)	0	38 (78%)
***Clinical***			
TB symptoms	1 (3%)*	6 (67%)	3 (6%)
BCG			
Scar present	23 (61%)	1 (11%)	21 (43%)
History but no scar	2 (5%)	1 (11%)	2 (4%)
No evidence of prior BCG	13 (34%)	7 (78%)	20 (41%)
Presence/absence of scar not recorded			6 (12%)
Tuberculin skin test †			
0–4 mm	0	0	40 (82%)
5–9 mm	3 (8%)	1 (11%)	8 (16%)
10–14 mm	12 (32%)	0	1 (2%)
≥15 mm	23 (60%)	7 (78%)	0
Induration median (range)	15 (8–37)	21 (6–24)	0 (0–10)
Chest radiograph			
Normal	35 (92%)	1 (11%)‡	19 (39%)
Abnormal	2 (5%) §	8 (89%)	1 (2%) ‖
Not done/unavailable	1 (3%)**	0	29 (59%)
***Laboratory***			
CD4 median (IQR) ††	40% (38–52%)	43% (30–56%)	40% (35–52%)

* Final diagnosis *Klebsiella pneumoniae* pneumonia; cough>2 weeks; sputum samples AFB negative.

† One child with TB disease did not have a TST.

‡ Extrapulmonary TB.

§ One child with evidence of prior TB; one child with right upper lobe opacification (*K. pneumoniae* on culture).

‖ Right upper lobe opacification (culture negative).

** Well, asymptomatic child; failed to attend for follow up CXR.

†† CD4 count available for 82 children.

The agreement between observers for the T-SPOT.*TB* assay results was high (interobserver reliability 96%). In four tests there was a discrepancy in assigning a result (one observer positive, two observers negative in each instance) with all four finally deemed negative by consensus. Although there were more indeterminate results with the T-SPOT.*TB* assay than with the QFT-IT assay overall (14/96 vs. 3/96, p = 0.009), the majority of indeterminate results for the T-SPOT.*TB* assay were attributable to potential laboratory error (Tables 2 and 3). Specifically, of the 14 indeterminate T-SPOT.*TB* assays, inadequate PBMC separation accounted for seven and cross-contamination occurred in two assays. Excluding these nine assays, the difference in the proportion of ‘true’ indeterminate results between the two assays was not statistically significant (3/96 vs. 5/87, p = 0.48).

**Table 2:** Results of QuantiFERON-TB gold in-Tube and T-SPOT.*TB* assays in each diagnostic category.

		QuantiFERON-TB gold In-Tube			T-SPOT.*TB*		
		Positive	Negative	Indeterminate	Positive	Negative	Indeterminate
All patients (n = 100)	Latent TB (n = 38)	18 (47%)	20 (53%)	(0)	15 (39%)	19 (50%)	4 (10%)*
	TB disease (n = 9)	8 (89%)	1 (11%)	0	9 (100%)	0	0
	Uninfected (n = 49)	2 (4%)	44 (90%)	3 (6%)†	1 (2%)	38 (78%)	10 (20%)‡
	No TST result (n = 4)	1 (25%)	3 (75%)	0	0	4 (100%)	0
Patients with TB contact (n = 44)	Latent TB (n = 22)	13 (59%)	9 (41%)	0	10 (46%)	10 (46%)	2 (9%) §
	TB disease (n = 9)	8 (89%)	1 (11%)	0	9 (100%)	0	0
	Uninfected (n = 11)	1 (9%)	8 (73%)	2 (18%) ‖	1 (9%)	5 (45%)	5 (45%)**
	No TST result (n = 2)	1 (50%)	1 (50%)	0	0	2 (100%)	0

* 3 inadequate PBMC; 1 cross contamination.

† 1 high nil control; 2 inadequate mitogen control.

‡ 3 high nil control; 4 inadequate PBMC; 2 technical; 1 cross contamination.

§ 1 inadequate PBMC; 1 cross contamination.

‖ 1 inadequate mitogen control; 1 high nil control.

** 3 inadequate PBMC; 1 technical; 1 high nil control.

**Table 3:** Comparison of the results of QuantiFERON-TB gold in-Tube and T-SPOT.*TB* assays.

			QuantiFERON-TB gold In-Tube		
			Positive	Negative	Indeterminate
T-SPOT.*TB*	All patients (n = 100)*	Positive	22 (22%)	2 (2%)	1 (1%)
		Negative	4 (4%)*	56 (55%)*	1 (1%)
		Indeterminate	3 (3%)	10 (10%)	1 (1%)
	Patients with latent TB infection (n = 38)	Positive	14 (37%)	1 (3%)	0
		Negative	2 (5%)	17 (45%)	0
		Indeterminate	2 (5%)	2 (5%)	0

* Includes children where TST result was unavailable.

κ (95% CI) for agreement between T-SPOT.TB and QuantiFERON-TB gold In-Tube in all patients was 0.83 (0.65–0.91) and in patients with latent TB infection was 0.82 (0.54–0.92).

The proportion of indeterminate results for both IGRA was higher in children younger than three years of age compared to those older than three years of age. Specifically, of 16 children younger than three years of age, two (12.5%) had an indeterminate QFT-IT assay result (one inadequate mitogen control, one high nil control) compared to one (1.1%) indeterminate result (inadequate mitogen control) in the 84 children older than three years of age (p = 0.06). Similarly, of the 16 children younger than three years of age, five (31.3%) had an indeterminate T.SPOT.TB assay result (four inadequate PBMC, one technical) compared to nine (10.7%) indeterminate results (three high nil control, two inadequate PBMC, two technical, two cross contamination) in the 84 children older than three years of age (p = 0.05).

When the assays both yielded interpretable (ie non-indeterminate) results, the overall agreement between QFT-IT and T-SPOT.*TB* was high (93% agreement, κ = 0.83, 95% CI 0.65–0.91). However, the results between the two IGRAs were discordant in six children (Table 3). Results were QFT-IT positive / T-SPOT.*TB* negative in four children (two with household TB contact and positive TST of 10 mm and 12 mm respectively, categorised as latent TB infection; one uninfected; one with a household TB contact in whom a TST result was unavailable). Results were QFT-IT negative / T-SPOT.*TB* positive in two children (one with household TB contact and a positive TST of 25 mm, categorised as latent TB infection; one with TB disease and a positive TST (22 mm)).

**Figure 1:**
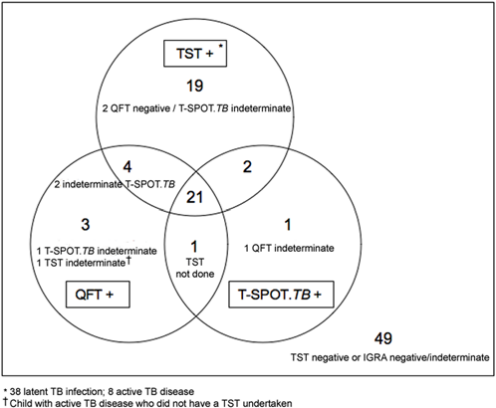
Venn diagram depicting agreement between tuberculin skin test, QuantiFERON-TB gold In Tube and T-SPOT.*TB* results for all children (n = 100).

There was only moderate agreement between TST and QFT-IT (75%, κ = 0.50, 95% CI 0.34–0.56) and between TST and T-SPOT.*TB* (75%, κ = 0.51, 95% CI 0.35–0.55) overall (Figure 1). The QFT-IT assay was positive in 18 (47%) and the T-SPOT.*TB* assay was positive in 15 (39%) of the 38 children with TST-defined latent TB infection (Table 2). Combining results from both IGRAs detected only one additional patient with TST-defined latent TB infection compared to QFT-IT alone, increasing the detection rate to 19/38 (50%). The results of both IGRAs were negative in 17 (45%) of the 38 children with TST-defined latent TB infection. Seven of these children had a TST induration ≥15 mm and eight had a TB contact (Table 4).

**Table 4:** Details of the results from children with latent TB infection and negative QuantiFERON-TB gold in tube or T.SPOT.TB results.

	IGRA result		TST induration (mm)		
			≥5 to 9	≥10 to 14	≥15
	QFT-IT	T-SPOT.*TB*	No. of children (No. non BCG immunised)		
All patients with latent TB infection (n = 22)	Negative	(1 positive)	3 (2)	7 (2)	10 (3)
	(2 positive)	Negative	3 (2)	9 (2)	7 (3)
	Both negative		3 (2)	7 (2)	7 (3)
	Either negative		3 (2)	9 (2)	10 (3)
Patients with latent TB infection and TB contact (n = 11)	Both negative		3 (2)	2 (2)	3 (1)
	Either negative		3 (2)	4 (2)	4 (1)

Three (6.1%) of the 49 children with a negative TST categorised as uninfected by study criteria were positive by at least one IGRA. These comprised a 9-year old (QFT-IT positive / T-SPOT.*TB* indeterminate (high nil control)) with no prior BCG immunisation, who was a household TB contact; a BCG-immunised 15-year old (QFT-IT positive / T-SPOT.*TB* negative) from a high TB prevalence country with no known TB contact; and a 2-year old (QFT-IT indeterminate / T-SPOT.*TB* positive) with no prior BCG immunisation, who was a household TB contact. Of the children with TB disease, results of the QFT-IT and T-SPOT.*TB* tests were positive in 8 (89%) and 9 (100%) children respectively.

**Figure 2:**
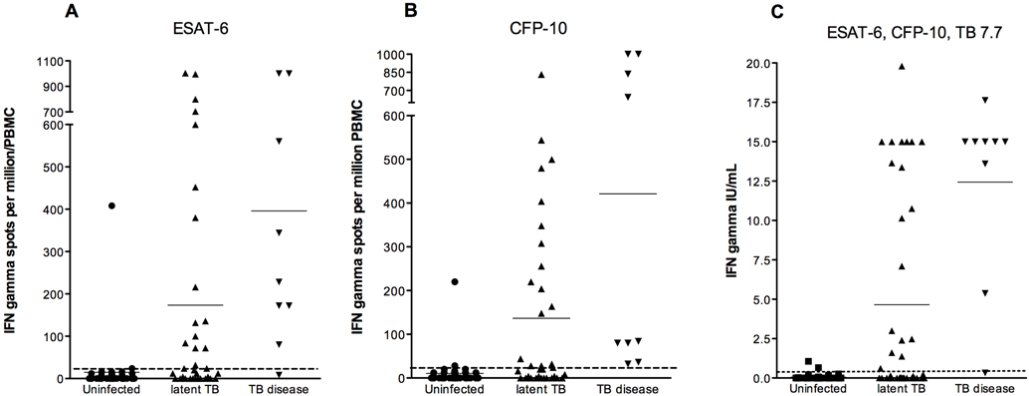
Results of T-SPOT.*TB* (panels A and B) and QuantiFERON-TB gold In Tube assays (panel C) according to tuberculin skin test-based diagnosis. The dotted line depicts the test cut-off value for a positive test.

The responses to the MTB-specific antigens for both IGRAs are shown in Figure 2. In the T-SPOT.*TB* assay, the mean (SEM) IFN-γ response to ESAT-6 and CFP-10 was higher in children with TB disease compared to those with latent TB infection (ESAT-6 396 (125.7) vs. 173.3 (51.1) spot forming cells per million PBMC (SFC/10^6^), p = 0.07; CFP-10 421.3 (146.2) vs. 136.5 (35.6) SFC/10^6^, p = 0.007). Similarly the IFN-γ response to ESAT-6 and CFP-10 was higher in children with latent TB infection compared to uninfected children (ESAT-6 173.3 (51.1) vs. 14.7 (10.4) SFCs/10^6^, p = 0.001; CFP-10 136.5 (35.6) vs. 10.4 (5.6) SFCs/10^6^, p = 0.0004). In the QFT-IT assay mean (SEM) IFN-γ responses to ESAT-6, CFP-10 and TB 7.7 were also significantly higher in children with TB disease compared to those with latent TB infection (12.44 (1.9) vs. 4.65 (1.06) IU/ml, p = 0.002). Similarly, children with latent TB infection had significantly higher mean IFN-γ responses than uninfected children (4.65 (1.06) vs. 0.06 (0.03) IU/ml, p<0.0001).

The influence of age, BCG scar, origin from country with high TB prevalence and TB contact on the results of TST and IGRAs was assessed. There was no significant difference in the median TST induration between BCG-immunised and BCG-non-immunised children overall or within the subgroup of children with latent TB infection (all children with TST >0 mm (n = 55): 13 mm vs. 17 mm; p = 0.25; children with latent TB infection (n = 38): 15 mm vs. 17 mm; p = 0.47). Among the 87 children with latent TB infection or who were uninfected, the mean age of those with a positive or negative QFT-IT (10.3 vs 9.2 years, p = 0.4) or T-SPOT.*TB* (10.3 vs 9.5 years, p = 0.54) assay was similar. Children with a TB contact were more likely than those without a TB contact to have a positive TST, a positive QFT-IT and a positive T-SPOT.*TB* (Table 5).

**Table 5:** The influence of TB contact on TST, QFT-IT and T-SPOT.*TB* results for the 87 children with latent TB infection (n = 38) or who were uninfected (n = 49).

		TB contact		Proportion positive with TB contact	Proportion negative without TB contact	LR (95% CI)
		Yes	No			
TST (cut off ≥5 mm)	Pos	22	25	22/33 (67%)	29/54 (54%)	1.4 (0.98–2.0)
	Neg	11	29			
TST (cut off ≥10 mm)	Pos	19	17	19/33 (58%)	37/54 (68%)	1.8 (1.1–2.8)
	Neg	14	37			
TST (cut off ≥15 mm	Pos	13	10	13/33 (39%)	44/54 (81%)	2.1 (1.0–4.2)
	Neg	20	44			
QFT-IT	Pos	14	6	14/31 (45%)	47/53 (89%)	3.9 (1.8–9.2)
	Neg	17	47			
T-SPOT.*TB*	Pos	11	5	11/26 (42%)	42/47 (89%)	3.9 (1.6–10.0)
	Neg	15	42			

In a multivariate linear regression analysis, age (p = 0.03) and TB contact (p<0.0001), but not BCG scar (p = 0.86) or origin from a country with high TB prevalence (p = 0.30), were significantly correlated with the magnitude of TST induration. In contrast, the only factor that significantly influenced the magnitude of the IFN-γ response in the IGRAs was TB contact (QFT-IT and T.SPOT.TB, p<0.0001). The proportion of children with a TB contact who had a positive TST (at both a 5 mm and a 10 mm cut-off) was higher than the proportion with a positive IGRA indicating a higher sensitivity for the TST in this setting (Table 5). In contrast, the IGRAs appeared to be more specific (with respect to TB contact) than the TST.

## Discussion

This is the largest study to date to compare the two currently available commercial IGRAs with TST for the diagnosis of latent TB infection specifically in children. The level of agreement between QFT-IT and T-SPOT.*TB* in our study (κ = 0.83) was higher than that observed in the studies by Arend *et al* (κ = 0.59) [8] and Leyten *et* al [9] (κ = 0.71). The infrequent occurrence of discordant results between the IGRAs suggests that either assay can be used depending on resources and availability. However, there was significant discordance between the results of TST and both IGRAs. This is important because IGRAs are increasingly considered potential replacements for TST for the detection of latent TB infection [10], [11]. Specifically, of 38 children with TST-defined latent TB infection, less than half had a positive QFT-IT or T-SPOT.*TB*. A critical question, with important implications for the future use and interpretation of IGRAs, is whether this is the result of false positive TST or false negative IGRA results [12].

**Figure 3:**
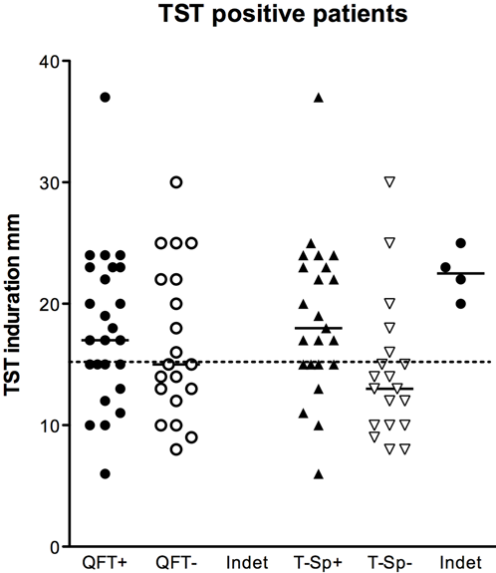
Relationship between results of QuantiFERON-TB gold In Tube and T-SPOT.*TB* assays and TST induration diameter in the 46 children with a positive tuberculin skin test (38 with TST-defined latent TB infection and 8 with TB disease). The broken line indicates a TST induration of 15 mm. QFT = QuantiFERON-TB gold In Tube, T-Sp = T-SPOT.*TB* assays, Indet = Indeterminate, TST = tuberculin skin test.

Several studies have questioned the sensitivity of IGRAs [8], [13], [14], [15], [16], [17], [18], [19], [20]. We previously found poor agreement between QFT-TB Gold (an earlier 2^nd^ generation QuantiFERON assay) and TST (κ = 0.3) for the diagnosis of latent TB infection [14]. In our previous study, a high proportion of children with a household TB contact positive by TST were negative by QFT-TB Gold and the assay was negative in almost half of the children with a TST >15 mm. Compared with our previous study, we found better agreement between TST and both QFT-IT and T-SPOT.*TB* although it is not clear whether this is clinically significant. For the QFT-IT assay, better agreement may be explained by the incorporation of an additional MTB-specific antigen (TB 7.7) conferring increased sensitivity [18]. However, the sensitivity of IGRAs for the detection of latent TB infection in children remains questionable with our finding that in children with TST indurations ≥15 mm who had latent TB infection, only 13/23 (57%) QFT-IT assays and 12/19 (63%) T-SPOT.*TB* assays were positive (Figure 3). Furthermore, in the 22 children with TST-defined latent TB infection with either a negative QFT-IT or a negative T-SPOT.*TB* result, almost a third had a TB contact and almost half had a TST ≥15 mm and, importantly, discordant results occurred even in the absence of BCG vaccination. Of note, there were three children (including one without prior BCG vaccination) with a TB contact and TST ≥15 mm in whom the results of both IGRAs were negative.

Our results are consistent with a recent large TB contact investigation in non-BCG vaccinated adults in which the QFT-IT and T-SPOT.*TB* assays were positive in only 42% and 51% respectively of those with a TST induration ≥15 mm [8]. Similarly, in another recent study, less than half of immigrants with a TST induration ≥15 mm were positive by QFT-TB Gold and the overall agreement between TST and QFT-TB Gold was poor (κ = 0.37) [21]. Another study in adults showed poor agreement between QFT-IT, an ELISPOT assay and a 6 day lymphocyte-stimulation assay [9]. Of 27 TST positive adults (mean induration 16 mm), 9 (33%) and 11 (46%) individuals tested positive by QFT-IT and ELISPOT respectively. One explanation for these results as well as the findings in our study is that discordant results are due to superior sensitivity of TST for the detection of latent TB infection revealing ‘false negative’ IGRA. However, the finding of three children in our study (two with a household TB contact) with a negative TST and at least one positive IGRA appears to be inconsistent with this explanation.

The alternate explanation for discordant TST and IGRA results is that IGRA have superior specificity and reveal ‘false positive’ TST results [22], [23], [24]. The lack of a gold standard for latent TB infection is a recognised inherent limitation of all studies that investigate the use of IGRAs for the detection of latent TB infection. Therefore estimating the sensitivity of any new test for latent TB infection is problematic. In the absence of a recognised gold standard, analysis of results with respect to TB contact history is one way to infer potential superiority of one test over another for the detection of latent TB infection. Although it is recognised that not all individuals exposed to a smear positive TB contact will subsequently become infected, this has become an accepted ‘gold standard’ on which to base comparative evaluations. Our finding that IGRAs have higher specificity than TST at any cut off with respect to TB contact history is consistent with the possibility that IGRAs have superior specificity for the detection of children with ‘true’ latent TB infection.

There are at least four further potential explanations for discordance between TST and IGRAs results. Firstly, in contrast to the TST which remains positive for a protracted period after past or cleared MTB infection [8], [25] recent evidence suggests that QFT-IT and T-SPOT.*TB* assays detect more recent or ongoing infection [26], [27]. This may be because the IGRAs predominantly detect effector MTB-specific T cells in an overnight stimulation assay whereas TST induration is measured at 48–72 hours allowing for the expansion of memory T cell populations [8], [13]. Interestingly a 6-day lymphocyte-stimulation test correlated better with TST than overnight IGRA in one study [9]. Secondly, IGRAs may revert to negative following clearance of TB infection [28]. Thirdly, prior sensitisation by non-tuberculosis mycobacteria, which is common in high TB prevalence countries, could lead to false positive TST results in some patients. In a recent study, 24% of children with non-tuberculosis mycobacteria had TST indurations >15 mm [29] and TST indurations >20 mm have been reported in this setting [30]. Lastly, the dose of the tuberculin PPD used in skin test reagents worldwide is not standardised. Though unlikely, the standard use of 10 IU of tuberculin PPD in Australia at the time of the study potentially resulted in more ‘false positive’ TST results.

The correct interpretation of a negative IGRA in a patient with a positive TST in routine clinical practice is challenging, particularly if the patient has a documented TB contact. In the absence of definitive evidence to confirm that discordant results are attributable to false positive TST results, our current practice is to offer isoniazid preventive treatment irrespective of IGRA result. However, a recent Japanese high school TB contact investigation provides preliminary evidence for withholding preventive treatment in individuals with TST positive / IGRA negative results [31]. Of 349 students in this study, 95 had a positive TST (defined as erythema ≥30 mm in those with prior BCG and contact with smear positive index case) of whom only four had a positive QFT-TB Gold assay result. Preventive treatment was offered to these four only, significantly reducing the number of students in whom chemoprophylaxis was prescribed. Importantly, no student has subsequently developed TB disease during a three and a half year follow up. However, this study had a relatively short follow up and does not address the issue of discordant results in young children in whom the risk of progression to TB disease may be higher. Also, until recently, it was not uncommon for individuals in Japan to have multiple BCG immunisations, potentially increasing the magnitude of TST responses. In addition, the measurement of erythema in defining a positive TST is unique to Japan and questions the applicability of these findings in countries where the measurement of induration is standard, despite limited evidence that there is good correlation between erythema and induration [32]. Therefore, before this approach can be considered safe, further, larger studies with longer follow-up are needed to investigate the natural history of children with TST positive / IGRA negative results and their risk of developing TB disease. Despite this, the UK National Institute for Clinical Excellence guidelines for the management of latent TB infection already recommend withholding preventive treatment in children over two years of age with a positive TST in whom the result of an IGRA is negative [33].

The higher rate of indeterminate results in the T-SPOT.*TB* assay compared with QFT-IT was attributable to a number of factors. The T-SPOT.*TB* is operationally more complex than the QFT-IT and 9 of the 14 indeterminate T-SPOT.*TB* results were attributable to potential laboratory error. The laboratory scientist undertaking the T-SPOT.*TB* assay underwent specific training prior to the study but given the relative complexity of the assay and the number of processing steps, the chance of a laboratory error occurring seems to be higher for the T-SPOT.*TB* assay. The indeterminate results potentially attributable to laboratory error were included in our analysis as they reflect real world practice. However, indeterminate results have been reported less frequently in the T-SPOT.*TB* assay than the QFT-IT assay in other studies [34]. The proportion of indeterminate QFT-IT assays was significantly less than the 17% reported in our previous study (p<0.001), the majority of which were due to a high nil control response [14], [35]. In the present study only one assay had a high nil control response as defined by the manufacturer's latest guidelines for test interpretation that allow a significantly higher background (nil control) response of up to 8 IU/ml. In our current study, 14% of children had a background IFN-γ response ≥1 IU/ml and 6% had a background ≥2 IU/mL. Several other studies have reported assays with high nil control responses [19], [36]. We have previously highlighted our concern about the validity of reporting assays with background levels ≥2 IU/mL [35]. For the T-SPOT.*TB* assay, four had background nil control responses ≥40 SFCs/10^6^. The cause of high nil control responses in IGRA warrants further investigation.

In conclusion we have shown high agreement between QFT-IT and T-SPOT.*TB* in children. Discordant results between TST and IGRAs are common (most often TST positive, IGRA negative) and highlight the need for further longitudinal studies to determine the negative predictive value of IGRAs and the validity of current recommendations for the investigation and treatment of latent TB infection in children.

## References

[1] (2000). Targeted tuberculin testing and treatment of latent tuberculosis infection.. Am J Respir Crit Care Med.

[2] Smieja MJ, Marchetti CA, Cook DJ, Smaill FM (2000). Isoniazid for preventing tuberculosis in non-HIV infected persons.. Cochrane Database Syst Rev.

[3] Pai M, Kalantri S, Dheda K (2006). New tools and emerging technologies for the diagnosis of tuberculosis: Part I. Latent tuberculosis.. Expert Rev Mol Diagn.

[4] Richeldi L (2006). An update on the diagnosis of tuberculosis infection.. Am J Respir Crit Care Med.

[5] Pai M, Dheda K, Cunningham J, Scano F, O'Brien R (2007). T-cell assays for the diagnosis of latent tuberculosis infection: moving the research agenda forward.. Lancet Infect Dis.

[6] Menzies D, Pai M, Comstock G (2007). Meta-analysis: new tests for the diagnosis of latent tuberculosis infection: areas of uncertainty and recommendations for research.. Ann Intern Med.

[7] Landis JR, Koch GG (1977). The measurement of observer agreement for categorical data.. Biometrics.

[8] Arend SM, Thijsen SF, Leyten EM, Bouwman JJ, Franken WP (2007). Comparison of two interferon-gamma assays and tuberculin skin test for tracing tuberculosis contacts.. Am J Respir Crit Care Med.

[9] Leyten EM, Arend SM, Prins C, Cobelens FG, Ottenhoff TH (2007). Discrepancy between Mycobacterium tuberculosis-specific interferon-{gamma} release assays using short versus prolonged in vitro incubation.. Clin Vaccine Immunol.

[10] Lalvani A (2007). Diagnosing tuberculosis infection in the 21st century: new tools to tackle an old enemy.. Chest.

[11] Mazurek GH, Jereb J, Lobue P, Iademarco MF, Metchock B (2005). Guidelines for using the QuantiFERON-TB Gold test for detecting Mycobacterium tuberculosis infection, United States.. MMWR Recomm Rep.

[12] Pai M, Menzies D (2007). The new IGRA and the old TST: making good use of disagreement.. Am J Respir Crit Care Med.

[13] Cehovin A, Cliff JM, Hill PC, Brookes RH, Dockrell HM (2007). Extended culture enhances sensitivity of a gamma interferon assay for latent Mycobacterium tuberculosis infection.. Clin Vaccine Immunol.

[14] Connell TG, Curtis N, Ranganathan SC, Buttery JP (2006). Performance of a whole blood interferon gamma assay for detecting latent infection with Mycobacterium tuberculosis in children.. Thorax.

[15] Dewan PK, Grinsdale J, Kawamura LM (2007). Low sensitivity of a whole-blood interferon-gamma release assay for detection of active tuberculosis.. Clin Infect Dis.

[16] Ferrara G, Losi M, Meacci M, Meccugni B, Piro R (2005). Routine Hospital Use of a New Commercial Whole Blood Interferon-{gamma} Assay for the Diagnosis of Tuberculosis Infection.. Am J Respir Crit Care Med.

[17] Hill PC, Brookes RH, Adetifa IMO, Fox A, Jackson-Sillah D (2006). Comparison of Enzyme-Linked Immunospot Assay and Tuberculin Skin Test in Healthy Children Exposed to Myocbacterium tuberculosis.. Pediatrics.

[18] Mahomed H, Hughes EJ, Hawkridge T, Minnies D, Simon E (2006). Comparison of mantoux skin test with three generations of a whole blood IFN-gamma assay for tuberculosis infection.. Int J Tuberc Lung Dis.

[19] Mazurek GH, Weis SE, Moonan PK, Daley CL, Bernardo J (2007). Prospective comparison of the tuberculin skin test and 2 whole-blood interferon-gamma release assays in persons with suspected tuberculosis.. Clin Infect Dis.

[20] Mazurek GH, Zajdowicz MJ, Hankinson AL, Costigan DJ, Toney SR (2007). Detection of Mycobacterium tuberculosis infection in United States Navy recruits using the tuberculin skin test or whole-blood interferon-gamma release assays.. Clin Infect Dis.

[21] Carvalho AC, Pezzoli MC, El-Hamad I, Arce P, Bigoni S (2007). QuantiFERON-TB Gold test in the identification of latent tuberculosis infection in immigrants.. J Infect.

[22] Ewer K, Deeks J, Alvarez L, Bryant G, Waller S (2003). Comparison of T-cell-based assay with tuberculin skin test for diagnosis of Mycobacterium tuberculosis infection in a school tuberculosis outbreak.. Lancet.

[23] Harada N, Nakajima Y, Higuchi K, Sekiya Y, Rothel J (2006). Screening for Tuberculosis Infection Using Whole-Blood Interferon- gamma and Mantoux Testing Among Japanese Healthcare Workers.. Infect Control Hosp Epidemiol.

[24] Lalvani A, Pathan AA, Durkan H, Wilkinson KA, Whelan A (2001). Enhanced contact tracing and spatial tracking of Mycobacterium tuberculosis infection by enumeration of antigen-specific T cells.. Lancet.

[25] Menzies D (1999). Interpretation of repeated tuberculin tests. Boosting, conversion, and reversion.. Am J Respir Crit Care Med.

[26] Mori T, Harada N, Higuchi K, Sekiya Y, Uchimura K (2007). Waning of the specific interferon-gamma response after years of tuberculosis infection.. Int J Tuberc Lung Dis.

[27] Suzuki K, Onozaki I, Shimura N, Harada N, Mori T (2004). QuantiFERON-TB-2nd Generation in the elderly persons.. Kekkaku.

[28] Hill PC, Brookes RH, Fox A, Jackson-Sillah D, Jeffries DJ (2007). Longitudinal assessment of an ELISPOT test for Mycobacterium tuberculosis infection.. PLoS Med.

[29] Detjen AK, Keil T, Roll S, Hauer B, Mauch H (2007). Interferon-gamma release assays improve the diagnosis of tuberculosis and nontuberculous mycobacterial disease in children in a country with a low incidence of tuberculosis.. Clin Infect Dis.

[30] Haimi-Cohen Y, Zeharia A, Mimouni M, Soukhman M, Amir J (2001). Skin indurations in response to tuberculin testing in patients with nontuberculous mycobacterial lymphadenitis.. Clin Infect Dis.

[31] Higuchi K, Harada N, Mori T, Sekiya Y (2007). Use of QuantiFERON-TB Gold to investigate tuberculosis contacts in a high school.. Respirology.

[32] Kimura M, Comstock GW, Mori T (2005). Comparison of erythema and induration as results of tuberculin tests.. Int J Tuberc Lung Dis.

[33] Taylor RE, Cant AJ, Clark JE (2007). Potential impact of NICE tuberculosis guidelines in paediatric TB screening.. Arch Dis Child.

[34] Ferrara G, Losi M, D'Amico R, Roversi P, Piro R (2006). Use in routine clinical practice of two commercial blood tests for diagnosis of infection with Mycobacterium tuberculosis: a prospective study.. Lancet.

[35] Curtis N, Connell T, Buttery JP, Ranganathan S (2006). Whole blood IFN-gamma assay for detecting TB in children. author reply.. Thorax.

[36] Nakaoka H, Lawson L, Bertel Squire S, Coulter B, Ravn P (2006). Risk for Tuberculosis among children.. Emerg Infect Dis.

